# Notes from the Field: Mortality Associated with Hurricane Matthew — United States, October 2016

**DOI:** 10.15585/mmwr.mm6605a3

**Published:** 2017-02-10

**Authors:** Alice Wang, Anindita Issa, Tesfaye Bayleyegn, Rebecca S. Noe, Christine Mullarkey, Julie Casani, Craig L. Nelson, Aaron Fleischauer, Kimberly D. Clement, Janet J. Hamilton, Christopher Harrison, Laura Edison, Kathrin Hobron, Katie M. Kurkjian, Ekta Choudhary, Amy Wolkin, Hurricane Matthew Incident Management System Team, CDC Emergency Operations Center

**Affiliations:** ^1^Epidemic Intelligence Service, CDC; ^2^Division of Environmental Hazards and Health Effects, National Center for Environmental Health, CDC; ^3^North Carolina Department of Health and Human Services; ^4^North Carolina Office of the Chief Medical Examiner; ^5^Career Epidemiology Field Officer Program, CDC; ^6^Florida Department of Health; ^7^Georgia Department of Public Health; ^8^Virginia Department of Health.

After 3 days as a Category 3 and 4 hurricane in Haiti and Bahamas, Hurricane Matthew moved along the coast of the southeastern United States during October 6−8, 2016 (*1*). Early on October 8, the storm made landfall southeast of McClellanville, South Carolina, as a Category 1 hurricane with sustained winds of approximately 75 mph, leading to massive coastal and inland flooding, particularly in North Carolina and South Carolina (*2*). Florida, Georgia, North Carolina, South Carolina, and Virginia made major disaster declarations; approximately 2 million persons were under evacuation orders in Florida, Georgia, North Carolina, and South Carolina (*3*). In response to the hurricane, CDC activated the Emergency Operations Center Incident Management System, tracked online media reports of Hurricane Matthew–associated deaths, and contacted states for confirmation of deaths. This report summarizes state-confirmed Hurricane Matthew–associated deaths that occurred during October 1−October 21 in Florida, Georgia, North Carolina, and South Carolina.

Forty-three hurricane-associated deaths were reported in four states; the median decedent age was 58 years (range = 9–92 years) (Table). Drowning was the most common cause of death, accounting for 23 (54%) deaths. Among all deaths, 26 (60%) occurred in North Carolina; 18 (69%) of these were drowning deaths associated with a motor vehicle. Twelve deaths occurred in Florida, including five that resulted from injuries during prestorm preparation or poststorm cleanup (e.g., a fall from a ladder or roof). A child’s death in Florida resulted from carbon monoxide poisoning related to indoor generator use.

**TABLE:** Characteristics of reported deaths related to Hurricane Matthew for all deaths including drowning — North Carolina, Florida, Georgia, and Virginia, October 2016

Characteristic	North Carolina (n = 26) No. (%)*	Florida (n = 12) No. (%)	Georgia (n = 3) No. (%)	Virginia (n = 2) No. (%)	Total (n = 43) No. (%)
**Sex**					
Male	18 (69)	9 (75)	3 (100)	2 (100)	**32 (74)**
Female	8 (31)	3 (25)	0	0	**11 (26)**
**Age group (yrs)**					
≤17	0	1 (8)	0	0	**1 (2)**
18–64	14 (54)	5 (42)	2 (67)	2 (100)	**23 (54**
≥65	11 (42)	6 (50)	1 (33)	0	**18 (42)**
Unknown	1 (4)	0	0	0	**1 (2)**
**Cause of death**					
Drowning	22 (85)	0	0	1 (50)	**23 (54)**
Trauma	2 (8)	8 (67)	3 (100)	1 (50)	**14 (33)**
Exacerbation of condition^†^	1 (4)	1 (8)	0	0	**2 (5)**
Electrocution	0	2 (17)	0	0	**2 (5)**
CO poisoning	0	1 (8)	0	0	**1 (2)**
Fire	1 (4)	0	0	0	**1 (2)**
**Directly related mechanism of death^§^**					
Vehicle drowning	18 (69)	0	0	0	**18 (42)**
Non-vehicle drowning	4 (15)	0	0	0	**5 (12)**
Tree-related trauma	1 (4)	2 (17)	2 (67)	0	**5 (12)**
**Indirectly related mechanism of death^§^**					
Vehicle crash injury	1 (4)	1 (8)	1 (33)	1 (50)	**4 (9)**
Preparation/repair injury	0	5 (42)	0	0	**5 (12)**
Electrocution	0	2 (17)	0	0	**2 (5)**
Medical exacerbation	1 (4)	1 (8)	0	0	**2 (5)**
CO poisoning	0	1 (8)	0	0	**1 (2)**
Fire	1 (4)	0	0	0	**1 (2)**

**Abbreviation:** CO = carbon monoxide.

* Percentages might not sum to 100% because of rounding.

^†^ Exacerbation of a person’s preexisting medical condition because of storm-related power failure.

^§^ A direct death is defined as a death caused by environmental forces of the hurricane and direct consequences of these forces, whereas an indirect death is caused by unsafe or unhealthy conditions as a result of loss/disruption of usual services, personal loss, or lifestyle disruption.

Despite public health warnings to avoid flood waters, among all 23 hurricane-related drownings, 18 deaths (78%) occurred in motor vehicles (e.g., vehicle driven into standing water, vehicle swept away by water, or person found in car). As little as 6 inches of water might result in loss of control of a vehicle, and 2 feet of water can carry most cars away (*4*). An evaluation of public health messages to drivers about avoiding flood waters might inform future prevention measures. Evaluation of the public’s reception and response to those messages, as well as an assessment of ascertainment of child deaths in disaster settings, might inform future prevention measures. Mortality surveillance after disasters plays a critical role in evaluating the causes, manners, and circumstances of deaths, and data can be used to guide prevention messages during the response and recovery period and to prevent deaths during future public health emergencies (*5*).

## References

[1] National Oceanic and Atmospheric Administration. Hurricane Matthew. Discussion number 26. Washington DC: US Department of Commerce, National Oceanic and Atmospheric Administration, National Hurricane Center; 2016. http://www.nhc.noaa.gov/archive/2016/al14/al142016.discus.026.shtml

[2] The Weather Channel. Hurricane Matthew recap: destruction from the Caribbean to the United States. Atlanta, GA: The Weather Company; 2016. https://weather.com/storms/hurricane/news/hurricane-matthew-bahamas-florida-georgia-carolinas-forecast

[3] Federal Emergency Management Agency. Hurricane Matthew. Washington DC: US Department of Homeland Security, Federal Emergency Management Agency; 2016. https://www.fema.gov/node/292516?utm_source=hp_promo&utm_medium=web&utm_campaign=femagov_hp

[4] CDC. Driving through water after a disaster. Atlanta, GA: US Department of Health and Human Services, CDC; 2012. https://www.cdc.gov/disasters/psa/driving.html

[5] CDC. Preliminary medical examiner reports of mortality associated with Hurricane Charley—Florida, 2004. MMWR Morb Mortal Wkly Rep 2004;53:835–7. 15371963

