# Erratum: Vol. 66, No. 5

**DOI:** 10.15585/mmwr.mm6613a5

**Published:** 2017-04-07

In the report “Notes from the Field: Mortality Associated with Hurricane Matthew — United States, October 2016” on page 145, the last sentence of the first paragraph should have read “This report summarizes state-confirmed Hurricane Matthew–associated deaths that occurred during October 1–October 21 in Florida, Georgia, North Carolina, **and Virginia**.”

